# Efficient Resource Scheduling for Multipath Retransmission over Industrial WSAN Systems

**DOI:** 10.3390/s19183927

**Published:** 2019-09-12

**Authors:** Hongchao Wang, Jian Ma, Dong Yang, Mikael Gidlund

**Affiliations:** 1 School of Electronic and Information Engineering, Beijing Jiaotong University, Beijing 100044, China; 2 Department of Information Systems and Technology, Mid Sweden University, Sundsvall 85170, Sweden

**Keywords:** Wireless Sensor and Actuator Networks (WSANs), multipath retransmission, resource scheduling, realtime wireless communication, monitoring and control system

## Abstract

With recent adoption of Wireless Sensor-Actuator Networks (WSANs) in industrial automation, wireless control systems have emerged as a frontier of industrial networks. Hence, it has been shown that existing standards and researches concentrate on the reliability and real-time performance of WSANs. The multipath retransmission scheme with multiple channels is a key approach to guarantee the deterministic wireless communication. However, the efficiency of resource scheduling is seldom considered in applications with diverse data sampling rates. In this paper, we propose an efficient resources scheduling algorithm for multipath retransmission in WSANs. The objective of our algorithm is to improve efficiency and schedulability for the use of slot and channel resources. In detail, the proposed algorithm uses the approaches of CCA (clear channel assessment)-Embedded slot and Multiple sinks with Rate Monotonic scheme (CEM-RM) to decrease the number of collisions. We have simulated and implemented our algorithm in hardware and verified its performance in a real industrial environment. The achieved results show that the proposed algorithm significantly improves the schedulability without trading off reliability and real-time performance.

## 1. Introduction

The process automation industry continuously works towards minimizing the cost of production and maintenance while improving the quality of their products. A step towards that goal is to utilize wireless technologies, which offer advantages such as less maintenance, more flexibility, and easy deployment in harsh industrial environments [1,2]. As one of the key technologies, industrial Wireless Sensor-Actuator Networks (WSANs) will play a vital role in the Industry 4.0 framework and Cyber Physical System (CPS) and will also be important for future smart factories and intelligent manufacturing systems [3]. However, harsh conditions, such as complicated environments and metal interference, result in more packet loss in WSAN transmissions compared with traditional wireless sensor networks [4]. Additionally, application-driven industrial communication increases the requirements of stringent reliability as well as time efficiency [5]. Within process automation, standards such as WirelessHART, ISA100.11a, and IEEE (Institute of Electrical and Electronics Engineers) 802.15.4e have been operational for nearly a decade, and there exists several researches that address support for emerging applications, such as data aggregation [6], control, and safety [7,8]. All of the above standards employ the Times-Slotted Channel Hopping (TSCH) Medium Access Control (MAC) protocol instead of pure contention-based MAC protocol Carrier-Sense Multiple Access with Collision Avoidance (CSMA/CA). The TSCH provides the capability of allocating a specific amount of bandwidth per node in a preknown pattern [9,10]. Moreover, channel hopping addresses unreliability caused by multipath fading and narrow-band external interference. However, these issued standards do not give specific link scheduling for efficient data transmission. An obstacle faced in guaranteeing deterministic communication in industrial environments, however, is that the sensor node hardware is usually equipped with an IEEE 802.15.4 compliant half-duplex single radio transceiver [11]. The resources such as slot and channel are restricted, since the transceiver supports only up to 16 different channels [12].

Communication ranges of most sensor devices are limited to short distances due to low power of transmission. Furthermore, obstacles in the wireless link or local interferences make single-hop topology WSANs difficult to deploy. Therefore, data packets originating from a source device are relayed to a destination device on end-to-end basis [13]. Thus, relaying packets in a multi-hop wireless network should be performed by reserving timeslots to provide on-time packet delivery. The unpredictable packet loss makes the retransmission scheme inevitably considered to achieve the desired levels of reliability. Multipath routing is one of the essential retransmission schemes to improve the robustness of end-to-end transmission in error-prone wireless communication environments [14].

However, multipath retransmission needs to be pre-allocated for more communication resources. Thus, the efficiency of resource scheduling is supposed to be considered. Moreover, the industrial WSAN is a technological paradigm that enables advanced and complex services by interconnecting a possibly large number of low-complexity resource-constrained embedded devices equipped with diverse sensors and actuators [15]. The incorporation that WSANs support the coexistence of different sampling rates is imperative for a better industrial solution. At such instances, resource scheduling needs to be considered for schedulability, since more collisions may exist in the process of resource allocation.

Addressing the above problems, this paper proposes an efficient resource scheduling of CCA (clear channel assessment)-Embedded slot and Multiple sinks with Rate Monotonic scheme (CEM-RM) for multipath retransmission. The proposed CEM-RM scheme aims at periodic sensors, and we assume that multiple sampling periods exist on scheduled sensor nodes. For event-driven sensors, the CEM-RM scheme is also applicable. However, the maximum delay of packet transmission is the length of a superframe. Since the time that events occur cannot be predicted accurately, the time of waiting on communication resources increases the total end-to-end delay. The scheduling of CEM-RM aims to deal with the problem of schedulability for multipath retransmission scheduling. Therefore, first, we assume that the multipath routing graph of all the nodes can be successfully constructed. Second, the IEEE 802.15.4-based radio transceivers support 16 channels on 2.4 GHz. The scheduling of CEM-RM needs multiple channels; thus, we assume that at least eight channels can be used for scheduling without interference. Finally the last assumption is that multiple sampling periods exist on scheduled industrial flows and that the minimum period should not be less than the transmission delay of nodes, which are at the greatest distance from the gateway. The main contributions of this paper are listed as follows:

1. We analyze the resource scheduling principle for the multipath retransmission scheme based on the WirelessHART standard, and we design a link release algorithm to cater the principle of routing order in data flow.
2. We use the approaches of multiple sinks, CCA-embedded timeslot, and rate monotonic scheme to respectively decrease the negative effects of collision, path crossing, and diverse period during resources scheduling. Based on the above approaches, we propose an algorithm (CEM-RM) to improve the schedulability of resources scheduling.
3. We finally simulate our proposed algorithm to prove the improvement of schedulability. Additionally, we implement a WSAN system with our proposed scheduling in a real factory. The practical experiments are conducted to prove the reliability and real-time performance.

The rest of the paper is organized as follows. Section 2 presents the related works, and Section 3 describes the system model and the principle of multipath retransmission scheme. Moreover, we present the constraints during resources allocation. In Section 4, we present the proposed Link Release algorithm and CEM-RM algorithm in more detail. The simulation and experimental results are illustrated in Section 5, and finally, we conclude our work in Section 6.

## 2. Related Works

The use of multiple channels in the TSCH MAC protocol enables benefits including diversity, network scalability, and optimized scheduling. In the last couple of years, a notable trend in multichannel MAC solutions can be seen in Reference [16]. Zhao et al. [17] present multichannel, TSCH-based source-aware scheduling schemes for WSNs. The algorithm benefits from multiple channels but fails to guarantee reliability. Dobslaw et al. [5] extend the SchedEx [18] scheme to a multichannel scenario by introducing scalable integration in existing schemes. The authors also claim to cut latencies around 20% in the schedules from ShedEx. Kim et al. [19] proposed a distributed solution based on multichannel allocation that achieves max-min fairness among multiple flows. However, the fairness of channel use in such approach needs to trade off throughput. Researches, such as References [20,21,22,23], deeply exploit the use of multiple channels. The approaches proposed in these literatures focus on decreasing the interferences while using multiple channels in their specific type of WSNs. Therefore, this paper also makes full use of available channels to optimize TSCH scheduling.

Multipath routing allows multiple streams of information from source to sink, which significantly increases the reliability of data reception, especially in industrial environments. Liew, Soung-Yue et al. [24] propose a complete process of channel assignments for adaptive and energy-efficient data collection protocol, which could schedule the node connectivity based on traffic load. Van Luu et al. [25] proposed a scheduling algorithm for multipath routing structures, the objective of which is to reduce the message complexity. Mo Sha et al. [26] propose a novel channel-hopping methods for the exchange of data packages, which could prevent links, sharing the same destination from using channels with strong correlations. Xi jin et al. [27] proposed a mixed criticality scheduling algorithm, which guarantees the real-time performance and reliability requirements of data flows with different levels of criticality. Through the empirical studies, the above researches also show that graph routing leads to significant improvements over source routing in terms of worst-case reliability, where graph routing protocol is a classical multipath transmission approach in the WirelessHART standard. However, none of these researches simultaneously consider the effect of collision, path crossing, and diverse period on TSCH scheduling, which also lacks concentration on schedulability in a large WSAN.

The gateway resides at the root of all graphs in a wireless network, and it can provide multiple network access points, which are named sink nodes in this paper. The responsibility of a sink node is to maintain the connection between wireless nodes and the gateway, where most researches regard a sink node as a gateway [28]. However, the sink node is a hotspot and experiences more link conflicts than other nodes do when we schedule a multi-hop routing graph. Due to the deadline of industrial data transmission, this characteristic seriously decreases the schedulable ratio, especially in larger networks. Therefore, this paper provides multiple sink nodes to avoid the link scheduling conflicts and to improve the use of communication resources. In Reference [29], the authors apply the theory of compressive sensing on a multi-sink wireless sensor network, which could improve the capability for data gathering. However, the presented architecture of the network is not complicated enough to support routing graphs. Multiple sinks are generally used in the distributed scenario to balance the traffic load, which is responsible for packet collection as a substation [30,31,32,33]. In this paper, we realize a multi-sink gateway to achieve a highly schedulable WSAN in a practical industrial application.

## 3. Network Model

### 3.1. Network Description

**Figure 1:**
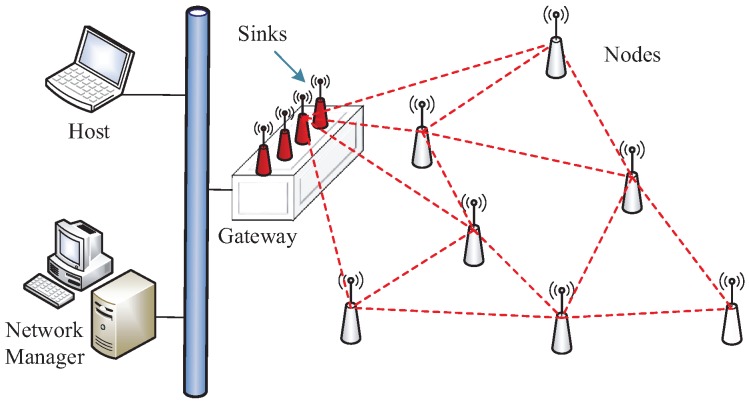
The structure of an industrial wireless sensor-actuator network (WSAN) system.

Deterministic communication heavily relies on resource scheduling management. Accordingly, network management techniques adapted for industrial wireless mesh are critical. This paper adopts centralized management to form a deterministic WSAN, the structure of which is shown in Figure 1. A network mainly consists of field nodes, multiple sinks, a gateway, and a centralized network manager. All field nodes operate over low-power radios compliant with the IEEE 802.15.4-2006 standard, which supports 16 channels in the 2.4 GHz ISM band. The TSCH MAC protocol is implemented in this paper to guarantee deterministic communication. One time slot is 10 ms long and allows for channel switching and the transmission of a single packet together with data-link acknowledgement (DL-ACK). Time slots can be either dedicated or shared, where the shared time slot is amended to CCA-Embedded Slot (CES) as proposed in Reference [34]. In a dedicated time slot (DTS), only a single transmitter–receiver pair can communicate on any given channel, thereby not allowing channel reuse. CES, in which multiple nodes are scheduled on the same channel, is mainly used for decreasing the use of slots for path crossing.

**Figure 2:**
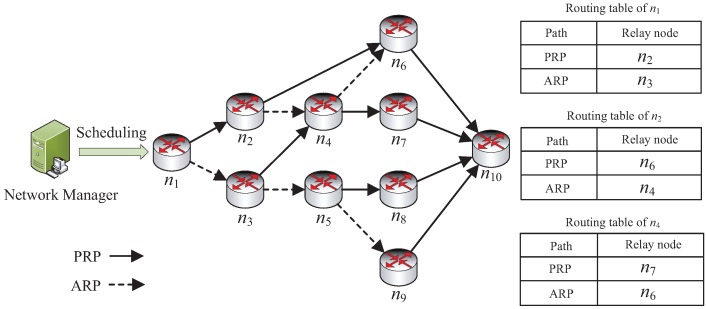
An example of the graph routing retransmission scheme.

To guarantee the reliability of the flow transmission, we adopt the graph routing retransmission approach advised in the WirelessHART standard. As shown in Figure 2, packets can be sent by a device which has two direct neighbors at least in a graph. One is the primary routing path (PRP), and another is the alternative routing path (ARP). Each type of path has a receiving node for relaying packets. A node should preferentially send packets along the PRP. If the transmission is failed in PRP, the node will change to using ARP. In this paper, we assume that the multipath graph is already generated by the network manager. Therefore, all the possible transmissions should be involved in a slot and channel scheduling. The scheduling of slot and channel for all packet flows also has some considerations and limitations in the following based on WirelessHART.

- The allocation of slot and channel based on graph routing is so that a device can send a packet at most three times, including the first transmission and two retransmissions. The first transmission and retransmission are along the PRP, and the second retransmission needs to change to be along the ARP, where the changes of link includes the neighbor and channel.
- According to the above consideration, each flow consists of dedicated and abundant transmissions, all which should be allocated for the slots and channels by the network manager. Moreover, each slot allocation should follow the routing orders, where the slot offset of next-hop transmission and retransmissions should not be allocated before the slots of the previous-hop transmission.

### 3.2. System Model

We describe the model of a WSAN, which consists of field devices, multiple sinks, a gateway, and a centralized network manager, as shown in Figure 1. We consider that the network can be modelled as a graph G=(N,L), in which the set $N=\{n_{1},n_{2},\cdots ,sk\}$ represents all the nodes, including field devices $n_{i}$ and sinks sk. *L* is the link set, where the element $l_{ij}$ in *L* indicates the connection state between the nodes $n_{i}$ and $n_{j}$. $l_{ij}=1$ means $n_{i}$ and $n_{j}$ can directly communicate with each other. $x(n_{i},n_{j})$ represents a transmission with the direction from node $n_{i}$ to $n_{j}$. Thus, for a link $l_{ij}$, $x_{t,c}\left(l_{ij}\right)$ represents the transmission allocated on the time slot *t* and channel offset *c*. The length of a time slot is denoted by κ. The actual channel can be calculated by (ASN+c) mod |C|, where ASN is the absolute counting of slot advance and *C* is an available channel set. Thus, the channel scheduling is only relevant to the assignment of channel offset. For example, $x_{t,c}\left(l_{ij}\right)=1$ represents that node $n_{i}$ transmits a packet to node $n_{j}$ using channel *c* at slot *t*, otherwise, $x_{t,c}\left(l_{ij}\right)=0$. Based on the above network model, all transmissions need to be scheduled by the TSCH MAC protocol. Thus, some basic constraints expressed by Equations (1)–(4) should be considered, which seriously influence the performance of transmission scheduling.

First, more than one transmissions cannot be occurred simultaneously in a certain slot and channel unless nodes use some contention-based mechanism. Otherwise, one transmission may generate wireless interference to interfere with the other transmissions.

(1) $$\sum_{n_{i}\in N}\sum_{n_{j}\in N}x_{t,c}\left(l_{ij}\right)\leq 1,\forall t,\forall c\in C$$

Second, the DL-ACK should be sent within one slot for reliability. Thus, a node can only transmit to one neighbor at any slot.

(2) $$\sum_{c\in C}\sum_{n_{j}\in N}x_{t,c}\left(l_{ij}\right)\leq 1,\forall t$$

Third, since a low-power node in the network only supports half-duplex wireless communication, obviously, it cannot be the transmitter and the receiver simultaneously.

(3) $$\sum_{c\in C}\sum_{n_{i}\in N}x_{t,c}\left(l_{ij}\right)+x_{t,c}\left(l_{ji}\right)\leq 1,\forall t$$

Finally, multichannel transmission can make full use of slot and channel resources; however, the number of available channels is limited. Thus, the number of transmissions cannot be beyond the number of available channels.

(4) $$\sum_{c\in C}\sum_{n_{i}\in N}\sum_{n_{j}\in N}x_{t,c}\left(l_{ij}\right)\leq \left|C\right|,\forall t$$

We consider all the end-to-end deliveries in a network as a flow set, which is denoted by $F=\{F_{0},F_{1},\cdots ,F_{\gamma }\}$. The number of flows that need to be scheduled is denoted as γ. A flow can be characterized by $F_{k}:\langle X_{k},E_{k},p_{k}^{\prime },\phi_{k}\rangle$, where $X_{k}=\left\{x^{k}\left(s_{1},d_{1}\right),\cdots ,x^{k}\left(s_{\tau },d_{\tau }\right),\cdots \right\}$ is the collection of all transmissions in a flow, $E_{k}$ is the collection of transmission release orders related to the routing order, and $\phi_{k}$ is the routing path from the first source node to the last destination node. $s_{\tau }$ and $d_{\tau }$ are the source node and the destination node in a transmission, respectively. One of the elements e(α,β) in $E_{k}$ represents that the transmission $x^{k}\left(s_{\beta },d_{\beta }\right)$ is released after all the transmissions $x^{k}\left(s_{\alpha },d_{\alpha }\right)$. Each $F_{k}$ periodically generates a packet with the period $p_{k}^{\prime }$, and the flow should be finished before its deadline $d_{k}$.

All the slots for a flow construct a superframe which is repeated periodically. The supported periods are defined as $2^{a}\cdot p_{m}$ based on WirelessHART, where *a* is an integer and $p_{m}$ is the minimum period among all flows. Without loss of generality, we transfer the period of all flows to the regular period through Equation (5), since it can let the scheduling fit with the data period better.

(5) $$p_{k}=p_{m}\cdot 2^{\left\lfloor log_{2}\frac{p_{k}^{\prime }}{p_{m}}\right\rfloor },p_{k}^{\prime }\geq p_{m},k=0,1,\cdots ,\gamma$$

For example, assuming the basic period is 20 ms, then the supported periods can be 20 ms, 40 ms, 80 ms, and so on. All the superframes constitute a hyperframe, the period of which is the maximum period among the superframes. The period is denoted as *T* and is expressed by Equation (6).

(6) $$T=max\{p_{k},k=0,1,\cdots ,\gamma \}$$

We use H[t][c] to denote which transmission is allocated on time slot *t* and channel *c*. The number of slots in a hyperframe is denoted by |H|=T/κ. As an example, if the first transmission of flow $F_{1}$ is allocated for slot 0 and channel offset 0, it can be expressed as $H\left[0\right]\left[0\right]=x^{1}\left(s_{1},d_{1}\right)$, otherwise, it is H[0][0]=EMPTY if there is no transmission allocated to H[0][0]. We assume that the deadline of a flow transmission is equal to its period in this paper. Thus, all the transmissions in a flow should be scheduled within the period, where the constraint can be expressed by Equation (7).

(7) $$t\leq \frac{p_{k}}{\kappa },\forall x^{k}\left(s_{\tau },n_{\tau }\right),k\in \left\{0,1,\cdots ,\gamma \right\}$$

## 4. The Proposed Scheduling

In this section, we introduce the CEM-RM algorithm for the multipath retransmission scheme based on graph routing in WirelessHART. We also present the proposed approaches to make the scheduling more efficient. The symbols and related functions used in the algorithms are listed in Table 1.

### 4.1. Transmissions Release Algorithm

As mentioned before, the multipath scheme requires that a packet on a device is transmitted three times at most, including initial transmission and two retransmissions. Thus, we first need to generate all the transmissions and their corresponding orders for a flow before allocating slots and channels. Algorithm 1 is the function of TransmissionRelease(F), which can generate all transmissions and transmission orders for a flow, according to a given graph *G*. Actually, the period *p* and the routing ϕ of the flow are already known and we mainly focus on producing the collections *X* and *E* to complete the flow. The initial transmission and the first retransmission use PRP, and the second retransmission uses ARP. A link in ϕ releases two transmissions if it is used for PRP; otherwise, it releases only one transmission for ARP. To obtain all the transmissions, we traverse all the links in ϕ from the source node to the destination node by a first in and first out (FIFO) pipe. Meanwhile, to generate the transmission order, we give each $n_{i}$ a transmission collection $X^{\prime }\left(n_{i}\right)$, which involves the already generated transmissions. The destination node of these transmissions is $n_{i}$. A node will be put into the FIFO and obliged to release the transmission until the number of transmissions in its collection $X^{\prime }\left(n_{i}\right)$ is equal to $D_{i}$. $D_{i}$ is the in-degree of $n_{i}$, which can be calculate by Equation (8).

(8) $$D_{i}=\sum l_{ji},l_{ji}\in \phi$$

**Figure 3:**
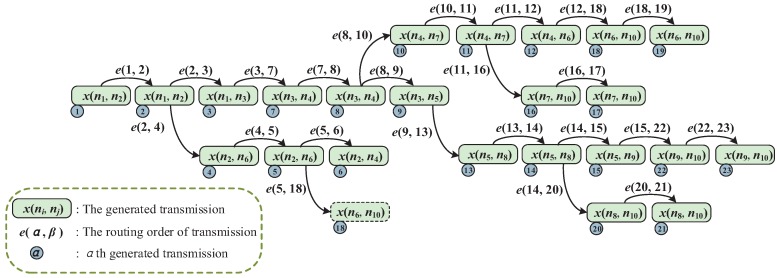
Generated transmissions and their transmission orders for an example flow.

To illustrate the function of TransmissionRelease(F), we consider the topology shown in Figure 2 as an example. The flow is generated by node $n_{1}$ and transmitted to node $n_{10}$. As shown in Figure 3, the generated transmissions in *X* are in released sequence after executing Algorithm 1. For example, the first released transmission is $x(n_{1},n_{2})$, and the fourth is $x(n_{2},n_{6})$. The transmissions in *X* also need sequence collection *E* to further explain their routing order, where e(2,4) represents that the 4th transmission $x(n_{2},n_{6})$ must occur after the second transmission $x(n_{1},n_{2})$. The transmissions without orders can occur simultaneously, which means they can be allocated for the same slot with different channels. We can see that the two adjacent transmissions may not be in continuous order, such as the 6th transmission $x(n_{2},n_{4})$ and 7th transmission $x(n_{3},n_{4})$. We also notice that *E* has two orders, e(5,18) and e(12,18), where the transmission $x(n_{6},n_{10})$ should go after the 5th and 12th transmissions. Actually, we only need to comply with the latter order, e(12,18), which should be considered in slot and channel allocation.


**Algorithm 1:**
TransmissionRelease(F)


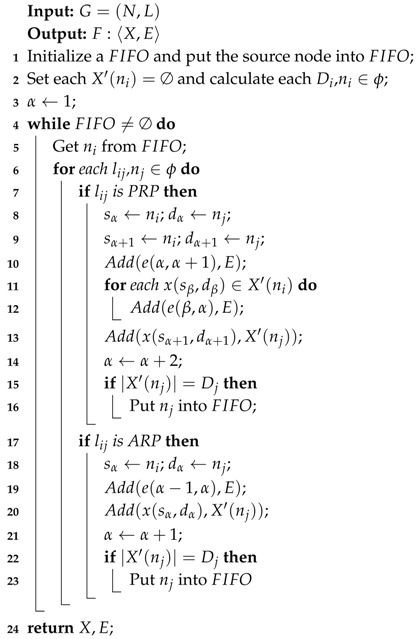



### 4.2. CEM-RM Algorithm

The proposed scheduling algorithm CEM-RM is shown in Algorithm 2. Considering decreasing the negative effect of the diverse period, path crossing, and conflict, the algorithm adopts three approaches to improve schedulability.

Assuming that $t_{\alpha }$ and $c_{\alpha }$ respectively represent the slot offset and channel offset allocated to the transmission $x^{k}\left(s_{\alpha },d_{\alpha }\right)$ within a hyperframe. For a flow $F_{k}$, if there is e(α,β), the slot allocation must satisfy $t_{\alpha }<t_{\beta }$. Meanwhile, all the slots $t_{\alpha }+j\cdot p_{k}\left(j\geq 0\right)$ are allocated to the transmission $x^{k}\left(s_{\alpha },d_{\alpha }\right)$ due to periodic characteristics. Since there exists node interference in the time slot, the objective of the scheduling is to avoid these interference. However, the slot allocation for transmissions has different periods. This causes more node interference, which reduces channel utilization. The given network graph is schedulable (SCH) when the slot allocation of all the flows satisfies the above constraints expressed by Equations (1)–(4) and (7); otherwise, it is unschedulable (UNSCH). As a classical scheduling strategy, rate monotonic (RM) policy preferentially assigns communication resources to the flow which possesses the shorter period [35]. We adopt RM policy as a basic strategy, where we sort all the flows in ascending period at the beginning (line 2).

**Figure 4:**
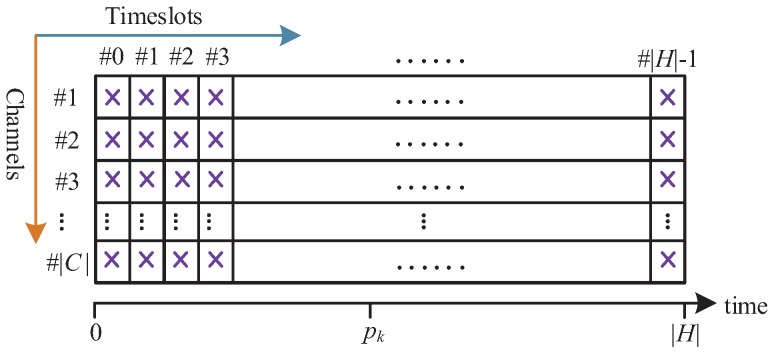
The communication resources for a hyperframe.

After individually tracing the RM-based scheduling, we notice that more transmissions need to be scheduled for a node that is close to the gateway, especially for the sink node. As shown in Figure 4, obviously, the sum of available communication resources is |H|·|C|, where we regard a slot with a channel as a communication resource. On the one hand, the generated transmissions of all flows cannot exceed the number of communication resources, which is expressed as follows:

(9) $$\sum_{k=0}^{\gamma }|X_{k}|\leq |H|\cdot |C|,F_{k}\in F$$

On the other hand, the number of available communication resources is $p_{k}\cdot \left|C\right|$ when the flow $F_{k}\in F$ is to be scheduled. However, some of these available resources are occupied by the previous k−1 allocated flows. The condition that there are enough available resources for $F_{k}$ is expressd as follows:

(10) $$\sum_{i=0}^{k-1}|X_{i}|\cdot \left\lfloor \frac{p_{k}}{p_{i}}\right\rfloor +|X_{k}|\leq \left\lfloor \frac{p_{k}}{\kappa }\right\rfloor \cdot \left|C\right|,\forall F_{k}\in F^{\prime }$$

**Algorithm 2:** CEM-RM

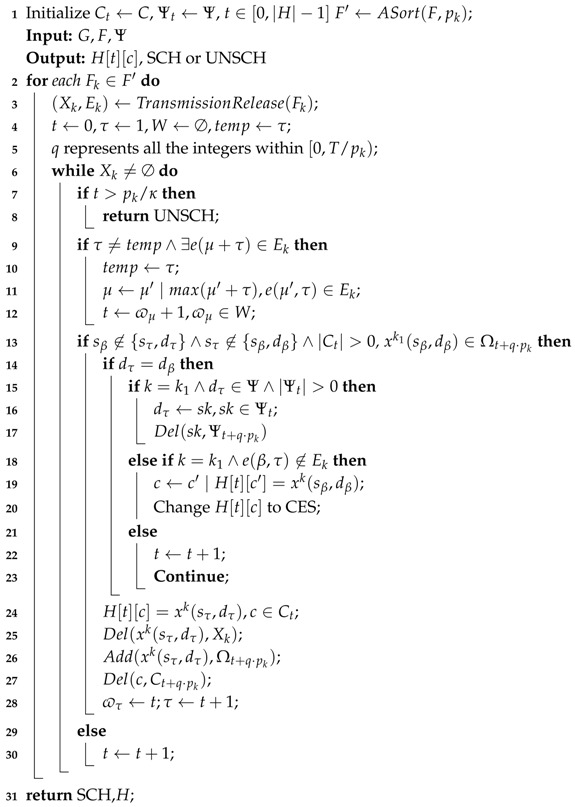



The available resources are becoming more inadequate with the increase of data flow. The later scheduled flows will meet more node interferences, especially at the end hop of a flow. This is because most transmissions share the sink node. A sink node cannot be involved in two transmissions simultaneously at a slot, which results in low schedulability. Moreover, the interference caused by one sink node makes it so that only one channel can be scheduled at the end of a hyperframe. Thus, we adopt multiple sinks to reduce the interference caused by a single sink node during hyperframe scheduling. Each slot possesses multiple available sinks to receive packets on different channels. In CEM-RM, a transmission may suffer interference at one slot, which is caused by a sink node. The algorithm will use the other available sink to avoid such interferences if at least one resource exists at the slot (line 19).

Furthermore, one key adjustment for the slot employed in this paper is that we amended the common shared slot to the CES. The multipath scheme provides an alternative relay node for possible retransmissions. Actually, a packet is finally routed along one of these paths. Redundant resources need to be assigned for avoiding conflict when the different routing paths share the same relay node. Thus, merging the resources for the overlapping paths is an effective approach. However, the conflict may be caused by the DL-ACK loss problem. A packet is simultaneously transmitted along more than one path if one of the transmissions only loses a DL-ACK. The packet is still transmitted along the original path. Considering this phenomenon deteriorates the reliability and costs extra energy, we adopt an efficient approach of the CES.

**Figure 5:**
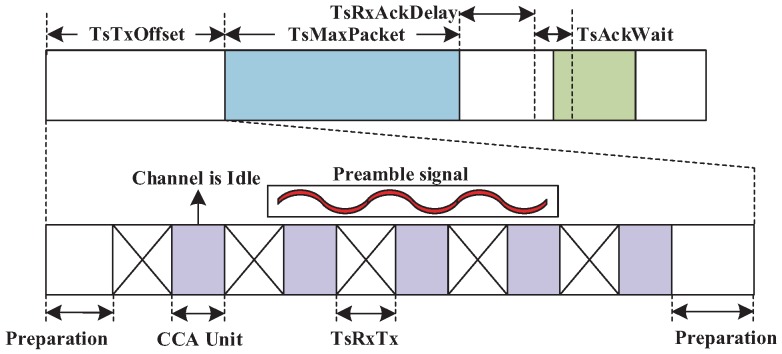
The structure of a CE (clear channel assessment-embedded) slot for the transmitting node.

At a CES, the timing structure of the transmitting node is shown in Figure 5 and the destination node still accords with the standard. The main adaptation is to add CCA units (128 μs) in the TsTxOffset, which is the 2120 ± 100 μs duration between the beginning of the slot and the start of the data frame transmission. We respectively reserve 200 μs and 340 μs at the beginning and end of TsTxOffset for some preparation of the slot and data frame. In CCA units, we adopt CCA mode 2 in IEEE 802.15.4 standard, where CCA shall report a busy medium upon the detection of a signal with the modulation and spreading characteristics of an IEEE 802.15.4 compliant signal. The maximum number of CCA units is 5 in this paper. Multiple source nodes will contend this slot by randomly selecting a CCA unit to detect the state of a channel. Once a source node detects that the channel is free, it sends a preamble signal to occupy the current slot during the remaining time TsTxOffset. Another source node will detect the preamble; then, it stops to perform CCA and to give up using the current slot. The radio chip may switch the state and channel in TsRxTx, which is less than 192 μs. We assume that there are *K* transmissions with different source nodes and the same destination node. The probability of successful contention is $\left(K/5\right)\cdot \sum_{j=0}^{4}{\left(j/5\right)}^{K-1},K\geq 0$ and enough to guarantee the reliability of the flow transmission, since the contention at CES occurs only when the DL-ACK is lost on the previous link. The use of CES is to decrease abundant slot allocation and to increase slot use, which also results in the increase of schedulable ratios.

With the use of the above three approaches, we designed the slot and channel allocation to avoid node interference. The following two situations should be solved by the above approaches when the allocation to a transmission $x^{k_{1}}\left(s_{\alpha },d_{\alpha }\right)$ meets the already allocated transmission $x^{k_{2}}\left(s_{\beta },d_{\beta }\right)$ in the same slot.

(1) According to constraints expressed by Equations (1)–(3), two different transmissions (α≠β) in a flow $\left(k_{1}=k_{2}\right)$ should be allocated for different slots if the source node of a transmission is the same as one of the nodes in another transmission. However, if $d_{\alpha }=d_{\beta }$ and two transmissions are allocated for the same slot exactly, those transmissions can use the same channel and the slot is changed into a CES. With the assumption of α>β, the condition can be expressed as follows:

$$\begin{array}{c} \mathrm{if}\;s_{\beta }\in \left\{s_{\alpha },d_{\alpha }\right\}\vee s_{\alpha }\in \left\{s_{\beta },d_{\beta }\right\}\;\mathrm{then}\\ \;(t_{\alpha }\neq t_{\beta })=1\\ \mathrm{else}\;\mathrm{if}\;d_{\alpha }\neq d_{\beta }\wedge e\left(\beta ,\alpha \right)\notin E_{k_{1}}\;\mathrm{then}\\ \;(\left(t_{\alpha }=t_{\beta }\right)\wedge \left(c_{\alpha }=c_{\beta }\right)\wedge \left(\mathrm{the}\;\mathrm{resource}\;\mathrm{is}\;\mathrm{CES}\right))=1\\ \mathrm{else}\;(\left(t_{\alpha }\neq t_{\beta }\right)\vee \left(c_{\alpha }\neq c_{\beta }\right))=1 \end{array}$$

(2) Accordingly, two transmissions in different flows $\left(k_{1}\neq k_{2}\right)$ also should be allocated for different slots if the source node of a transmission is the same as one of the nodes in another transmission. Also, if $d_{\alpha }=d_{\beta }\neq sk$, CES cannot be used for the transmissions in different flows. However, if $d_{\alpha }=d_{\beta }=sk$, we can change $d_{\alpha }$ to one of the other available sink nodes. In this scenario, the above two transmissions can be allocated at the same slot with different channels. With the assumption of $p_{k_{1}}>p_{k_{2}}$, *q* represents all integers within $\left[0,p_{k_{1}}/p_{k_{2}}\right)$. Therefore, the condition can be expressed as follows:

$$\begin{array}{c} \mathrm{if}\;s_{\beta }\in \left\{s_{\alpha },d_{\alpha }\right\}\vee s_{\alpha }\in \left\{s_{\beta },d_{\beta }\right\}\;\mathrm{then}\\ \;(t_{\alpha }\neq t_{\beta }+q\cdot p_{k_{2}})=1\\ \mathrm{else}\;\mathrm{if}\;d_{\alpha }=d_{\beta }=sk\wedge \left|\Psi_{t}\right|>0\;\mathrm{then}\\ \;(\left(t_{\alpha }=t_{\beta }+q\cdot p_{k_{2}}\right)\wedge \left(c_{\alpha }\neq c_{\beta }\right))=1\\ \mathrm{else}\;(\left(t_{\alpha }\neq t_{\beta }+q\cdot p_{k_{2}}\right)\vee \left(c_{\alpha }\neq c_{\beta }\right))=1 \end{array}$$

The above two conditions represent the situations of node interference and scheduling interference in slot and channel scheduling. The difference between the designed algorithm and RM algorithms is that we assign the communication resources by the order of each flow, instead of the slot sequence. Thus, we traverse all the transmissions in each period-sorted flow (lines 3 and 6) and assign the slots and channels to each transmission. To avoid interference, the scheduling should be adjusted when the allocation meets these conditions. We add all assigned transmissions at time slot *t* into $\Omega_{t}$. We define $C_{t}$ and $\Psi_{t}$ as the collections of available channels and sinks at time slot *t*, respectively. Their elements will be removed by the Del() function when the corresponding channels or sinks have already assigned. Moreover, $\varpi_{\tau }$ is used to record the slot offset allocated to the transmission τ in every flow, and it is responsible for finding the earliest available slot which does not break the routing order (lines 11–13). There could be several transmissions at a CES. Thus, changing a normal slot to a CES means that the slot expands for multiple transmissions. With the above considerations, we can finally obtain the scheduling table *H* when all transmissions are scheduled within their period.

In the algorithm, we define $|N_{F}|$ and $|X_{F}|$ as the maximum number of nodes and transmissions, respectively, and they are all related to the length of the flow. For example, for a four-hop flow in a routing tree topology, $|N_{F}|$ is less than 24. All the nodes have a PRP except the sink node and have an ARP except the first-hop nodes and the sink node. Thus, $|X_{F}|$ is less than $2\times \left(2^{4}-1\right)+1\times \left(2^{3}-1\right)=37$. The number of iterations of the “**for**” loop in line 3 and the “**while**” loop in line 6 are O(γ) and O(|H|), respectively. Moreover, the time complexities in line 2, line 4, line 11, and line 14 are O(γlog(γ)), $O(|N_{F}{|}^{3})$, $O(|X_{F}|)$, and $O(T/p_{m})$, respectively. Therefore, the time complexity in a worst case is $O(\gamma log\left(\gamma \right)+\gamma (|N_{F}{|}^{3}+\left|H|\right|X_{F}|+|H|\cdot T/p_{m}))$. In practicality, the periods of different applications could not have much difference; thus, $\left|H\right|\cdot T/p_{m}$ could be ignored.

## 5. Simulations and Experiments

In this section, we evaluate the performance of the proposed algorithm by comparing with two widely used real-time scheduling policies, RM and Least Laxity First (LLF) [36]. RM schedules a transmission based on the packet’s absolute deadline, while LLF schedules a transmission based on the packet’s laxity, which is defined as the transmission’s deadline minus remaining transmission time. The schedules of individual RM and LLF are not suitable with our applications due to the quantity of nodes and period. Therefore, we also add multiple sinks into each compared scheduling policy for fairness. Thus, we respectively denote the two schedules as M-RM and M-LLF in the following simulations and experiments.

Different topology structures need different quantities of communication resources. Thus, four kinds of topology structures are generated for comparison, which are denoted as Tp1, Tp2, Tp3, and Tp4. We assume that the furthest leaf node is four hops away from the gateway in all topology structures. In industrial wireless networks, since there is the requirement of reliable and real-time performance, on the one hand, the big hop size can increase the rate of packet loss and, on the other hand, the large network cannot cater the demand of transmission deadlines. In our experience, four hops can overlap the general factory. Meanwhile, four hops can exactly overlap the experimental factory in this paper. The difference between each topology structure is that the number of nodes on each hop is generated according to different probabilities. For example, to generate one of the Tp1 topologies, we first traverse all the nodes, where the total number of nodes is determined previously. The traverse approach stipulates that a node has a 0.3 probability of being on the first hop, a 0.3 probability of being on the second, a 0.3 probability of being on the third, and a 0.1 probability of being on the fourth. Then, each node on the lower hop randomly selects two higher-level nodes as their parents to be the major path and redundant path. Thus, all the topologies of the Tp1 structure may be different from each other. Without loss of generality, we will stochastically generate enough topologies of every kind of structure. With the same method, the other kinds of topologies are generated according to the corresponding probabilities, which are listed in Table 2.

### 5.1. Simulations

Our simulations are presented to demonstrate the schedulability and effectiveness of our scheduling. The algorithms have been written in C language, and the simulations have been performed on a Windows machine with a 3.1-GHz Inter Core 3 processor. The following metrics are used for performance analysis: (a) Schedulable ratio is measured as the percentage of test cases for which a scheduling policy is able to schedule all transmissions. (b) Scheduling time is the total time required to successfully make a complete hyperframe for all transmissions. (c) Average normalized bandwidth is the ratio between the number of allocated resources and the total resources within a hyperframe after scheduling. In simulations, we assume that every node generates a packet periodically and sends it to the gateway along the routing graph. A packet is regarded as a flow without aggregation. The period is randomly selected in the set $\left\{p_{m}\cdot 2^{a}|\forall a\in \left[0,b\right]\right\}$, where *b* is the maximum exponent. A packet belonging to a fourth-hop node needs at most 22 transmissions using the scheduling of multipath scheme; thus, three values of $p_{m}\left(0.25\;s,0.5\;s,1\;s\right)$ are tested in the simulations.

**Figure 6:**
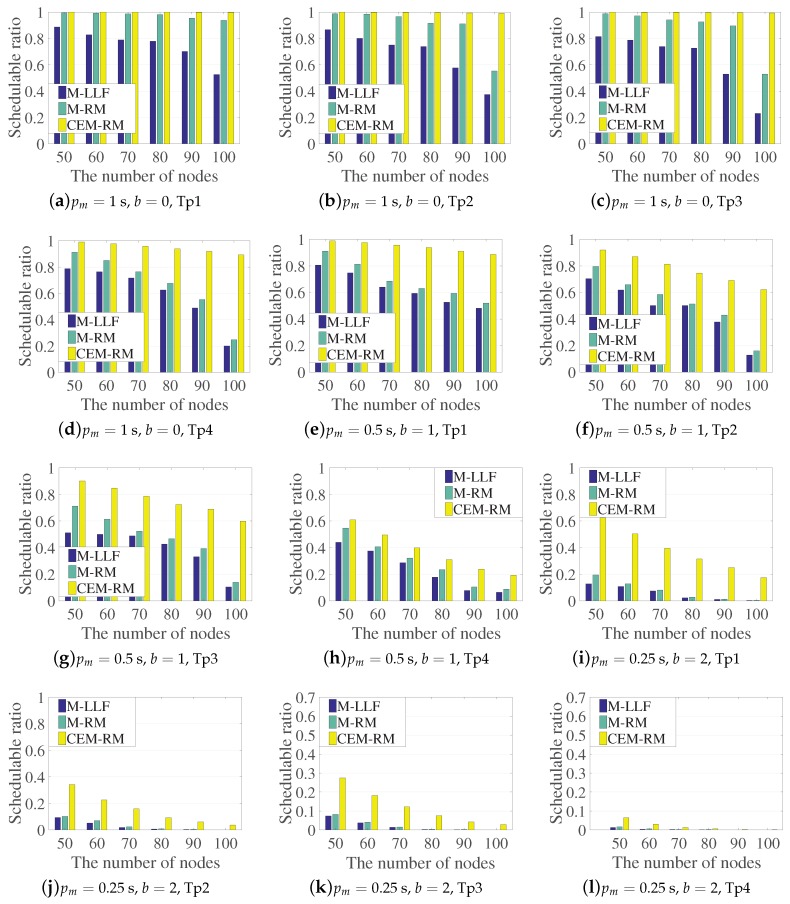
A comparison of the schedulable ratios of the three algorithms with different periods and topologies.

Figure 6 shows the schedulable ratio of the three scheduling policies with different parameter configurations. The number of nodes ranges from 50 to 100. We randomly generate 8000 cases for each node quantity and use the three algorithms to schedule the generated topologies separately. The number of available channels is set to be the maximum, 16, for a better comparison, since several cases using less channels are hardly scheduled with the existence of diverse periods. In Figure 6a–d, the three algorithms, which execute on each type of topology with $p_{m}=1$ s and b=0, can successfully schedule the majority of graphs. Obviously, the schedulable ratio drops in pace with the increase of nodes; however, we can see that our proposed scheduling is almost unaffected with these configurations. With the period shortened and diversified, the number of schedulable graphs decreases dramatically. Figure 6e–h and Figure 6i–l show the results under the period configurations of $p_{m}=0.5$ s, b=1 and $p_{m}=0.25$ s, b=2, respectively. They clearly indicate that the schedulable ratio of CEM-RM is higher than that of M-LLF and M-RM. Especially, increasing the number of nodes makes the gaps between CEM-RM and the others even bigger.

**Figure 7:**
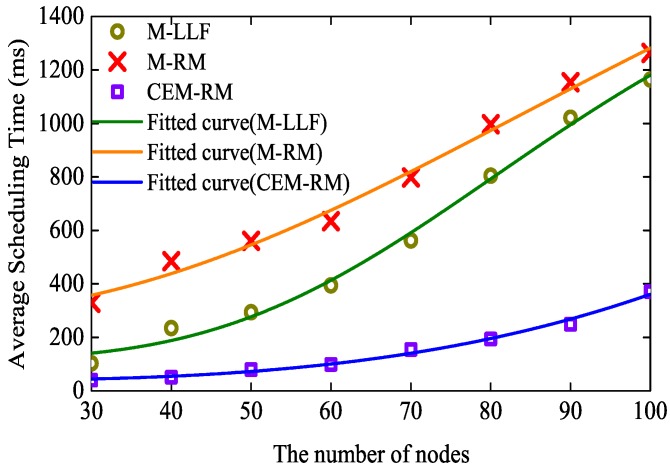
Comparison of scheduling time of the three algorithms.

The execution time of a scheduling can affect the performance of network formation and rescheduling; thus, we have measured the average run time of the three algorithms. Each test with a different number of nodes and algorithms includes 100 successfully scheduled graphs. To guarantee that the tested graphs are more schedulable, we set $p_{m}=0.5$ s and b=1 in all tests. As displayed in Figure 7, the markers represent the average time of different algorithms with different numbers of nodes and the lines show the trend of scheduling time as the number of nodes is increased. We can obtain that the average execution times of M-LLF and M-RM are lower than 1264 ms, while the time of our proposed scheduling is lower than 371 ms. The results indicate that CEM-RM is better for the performance improvement of network formation or rescheduling.

**Figure 8:**
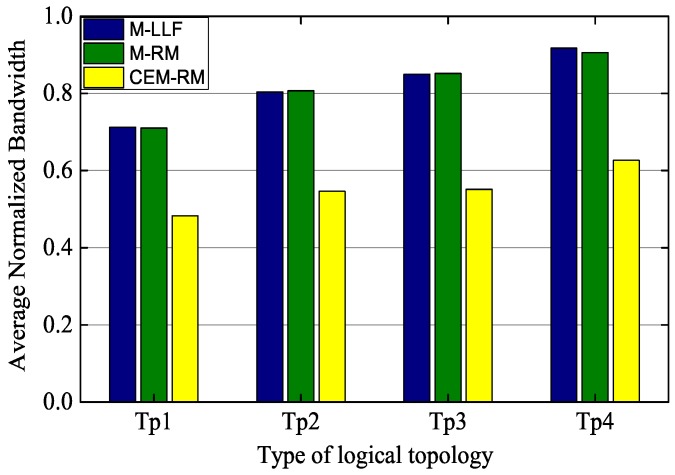
Comparison of average normalized bandwidth.

Moreover, Figure 8 shows the average requirement of the wireless bandwidth for our scheduling and for the others. The normalized bandwidth is calculated by the ratio between the number of allocated resources and total resources in a hyperframe. We also select the successfully scheduled hyperframe with 100 nodes and the configuration of $p_{m}=0.5$ s, b=1. As can be seen, the average normalized bandwidths of Tp4 of M-LLF and M-RM are 0.918 and 0.906, respectively, which are similar to each other, while CEM-RM only requires 0.627. The same conclusion also applies to other types of topologies. This is because the number of transmissions is determined by a graph in M-LLR and M-RM. However, CEM-RM adopts CES to solve the problem of overlapping paths and to reduce the phenomenon of DL-ACK loss, which can decrease the number of dedicated slots additionally. The remaining unscheduled resources can be allocated for network management to improve network stability.

### 5.2. Experiments

**Figure 9:**
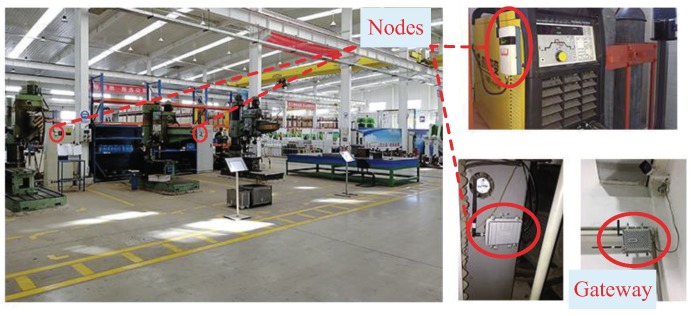
Experimental environment and deployment of field devices.

We also test the network performance of the scheduling policies in practice, where the performance includes the reliability and end-to-end packet delay. Since the scheduling of M-LLF and M-RM follows the basic policy (the number of retransmissions) in WirelessHART, their reliability and end-to-end packet delay are only affected by the channel condition. Thus, we make comparisons only between our scheduling and M-RM. The algorithms are executed on a developed WSAN system, which is compatible with WirelessHART and also supports our applications. As shown in Figure 9, the experiments are conducted in a 50 × 30 m^2^ factory in which the packet error rate is about 8% mainly caused by metal and electromagnetic interference.

**Figure 10:**
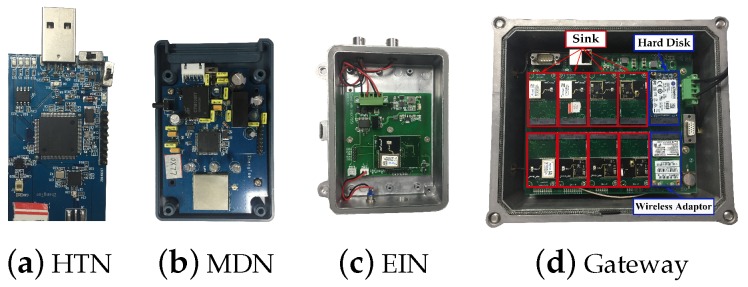
The hardware of wireless nodes and gateway.

**Figure 11:**
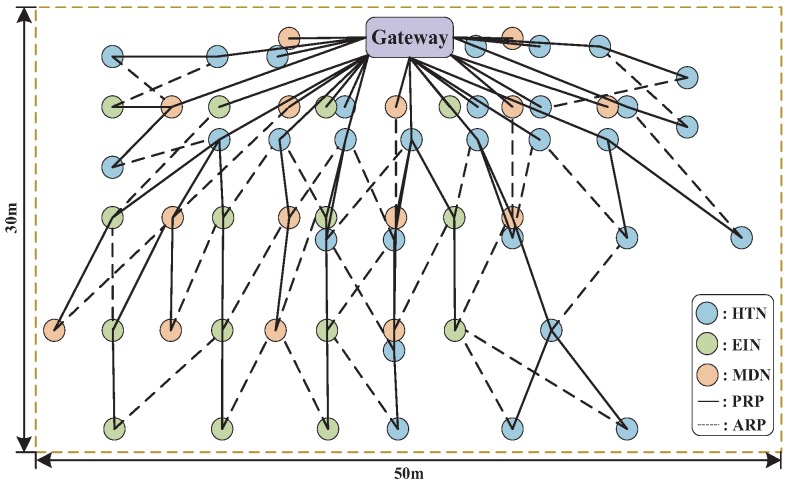
Experiment topology.

Our wireless nodes used to collect corresponding information are implemented on a LPC1769 mote with an AT86rf231 radio transceiver. In reality, we have three kinds of information to be collected, which are temperature and humidity (HT) with a 1-s period, electrical information (EI) with a 0.5-s period, and machine data (MD) with a 0.25-s period. Each kind of information is collected by different versions of wireless node, respectively shown in Figure 10a–c. A total of 60 nodes consist of 30 HT nodes (HTN), 15 EI nodes (EIN), and 15 MD nodes (MDN). The information of MD is collected through a CAN (Controller Area Network) bus, and the EI is transmitted by RS485. The radio transmission power is 3 dBm, and we use 10 of the available channels with lower packet error rates. The multi-sink-based gateway with 8 sinks, a hard disk, and a wireless adaptor is shown in Figure 10d. The communication between multiple sinks and the gateway is constructed by USB port. The metal case is used to avoid dust and water to increase the lifetime. The generated topology is shown in Figure 11, and the remaining experimental parameters are listed in Table 3.

**Figure 12:**
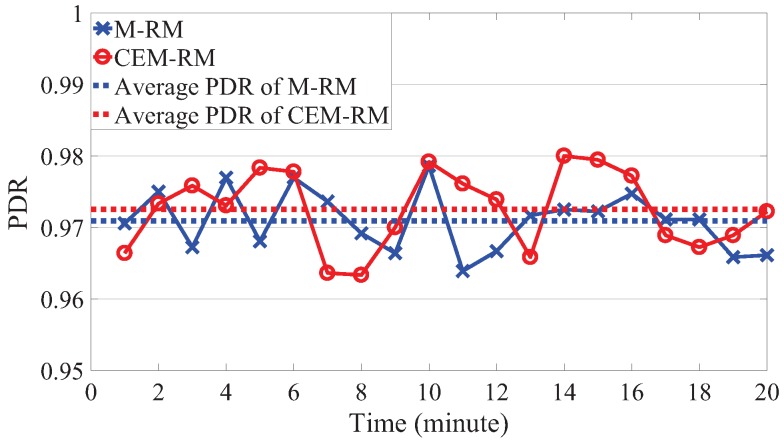
The packet delivery ratios of CEM-RM and M-RM.

On the one hand, we first measure the packet delivery ratio (PDR) of M-RM and CEM-RM, as shown in Figure 12. The PDR represents the ratio between the number of packets that succeed in arriving to the gateway on time and the total number of generated packets. Since the period of packet generation is known previously, we can get the total number of packets of each node. Then, we gather the statistic of the number of packets that successfully reach the gateway within their own deadline. An experiment remains 20 min, and we assess the PDR every minute. The average PDRs of M-RM and CEM-RM for 20 min are 0.971 and 0.973, respectively. We can observe that their PDR for every minute vibrates over their average values because the condition of channels may change with time. The jitter of our CEM-RM is a little higher than that of M-RM, where the minute-PDR standard variations of M-RM and CEM-RM are 0.0042 and 0.0055, respectively. The time-varying channel has an effect on the adopted CES to a certain extent. However, the average PDR is mainly the same, which means that the schedulability of our proposed scheduling is improved but does not sacrifice reliability.

**Figure 13:**
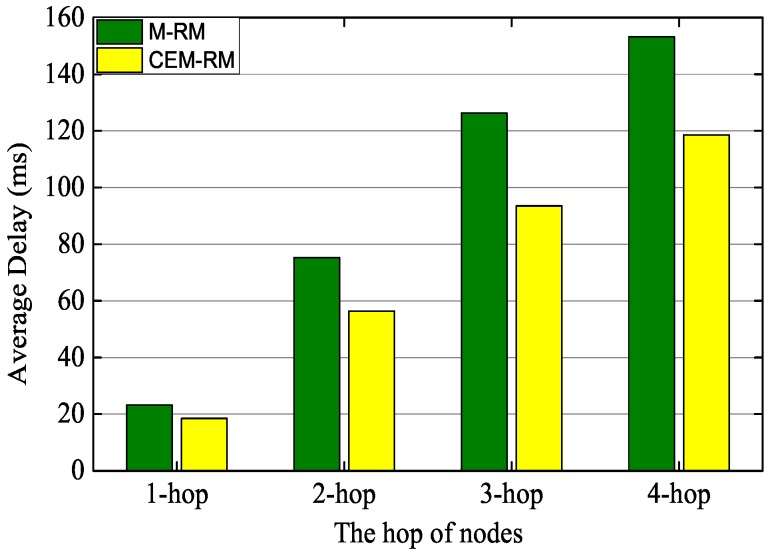
Comparison of average end-to-end delay between CEM-RM and M-RM.

On the other hand, we measured the delay of end-to-end delivery between each hop node and the gateway. The starting time of a packet is recorded at the time of its first transmission, while the ending time is recorded at the packet reception in the gateway. Figure 13 shows the comparison of average end-to-end delays between M-RM and CEM-RM. Obviously, the delay of CEM-RM is generally lower than that of M-RM. Moreover, as the routing hop increased, the average delay of our proposed scheduling was much lower than that of M-RM. For the packets of four-hop nodes, the average delay of our scheduling is about 32 ms lower than that of M-RM. The use of CES and multiple sinks effectively decreases the end-to-end delay, and CES can also reduce the impact of the DL-ACK loss phenomenon. The experimental results roughly indicate that our proposed scheduling not only improves the efficiency of scheduling but also decreases the end-to-end delay.

## 6. Conclusions

In this paper, we study the problem of how to improve the schedulability of communication resources in industrial WSANs. Existing centralized approaches for multipath retransmission scheme do not consider schedulability in the coexistence of diverse industrial applications. In this paper, we propose a resources-scheduling algorithm based on the multipath retransmission scheme in the WirelessHART standard. We use the approaches of RM policy, CES, and multiple sinks to reduce the negative impact of diverse periods and to avoid node interference as far as possible. By simulation and implementation in hardware, we compare our proposed CEM-RM algorithm with M-LLF and M-RM scheduling policies. The achieved results show that our proposed solution outperforms the aforementioned scheduling policies in terms of less use of communication resources, end-to-end delay, and increase in schedulable ratio without jeopardizing network reliability and timeliness of data packets.

## Figures and Tables

**Table 1:** Symbols and functions.

**Symbols**	**Description**
$D_{i}$	the number of links $l_{ji}$ in a subgraph, where the node $n_{i}$ is the destination node.
H[t][c]	the transmission allocated for time slot *t* and channel *c*
*W*	the collection of time slots, which are already allocated for transmission τ, where its element is $\varpi_{\tau }$
$C_{t}$	the collection of available channels at time slot *t*
$\Omega_{t}$	the collection of all the transmissions allocated to time slot *t*
$\Psi_{t}$	the collection of available sinks at time slot *t*
$|C_{t}|$, $|\Psi_{t}|$	the number of corresponding elements in the collection
**Functions**	**Description**
Add(y,Y)	the function responsible for adding an element *y* into the collection *Y*
Del(y,Y)	the function responsible for deleting an element *y* into the collection *Y*
ASort(Y,z)	the function responsible for sorting all the elements in *Y* in ascending order, according to feature *z*

**Table 2:** The proportions of different types of topology.

Type of Topology	Probability			
	1st Hop	2nd Hop	3rd Hop	4th Hop
Tp1	0.5	0.3	0.1	0.1
Tp2	0.5	0.2	0.2	0.1
Tp3	0.4	0.3	0.2	0.1
Tp4	0.3	0.3	0.3	0.1

**Table 3:** Experiment parameters.

Parameters	Description
The number of nodes	60
The number of nodes in each hop	1-hop:26, 2-hop:18, 3-hop:10, 4-hop:6
Transmission rate	250 kb/s
Transmission rate	3 dBm
The number of CCA units at a CES	5
Maximum frame retries	3
Packet length	80 bytes
